# Neuronal Mitophagy in Neurodegenerative Diseases

**DOI:** 10.3389/fnmol.2017.00064

**Published:** 2017-03-08

**Authors:** Marta Martinez-Vicente

**Affiliations:** ^1^Neurodegenerative Diseases Research Group, Vall d’Hebron Research InstituteBarcelona, Spain; ^2^Autonomous University of Barcelona (UAB)Barcelona, Spain; ^3^Centro de Investigación Biomédica en Red sobre Enfermedades Neurodegenerativas (CIBERNED)Barcelona, Spain

**Keywords:** mitophagy, autophagy, neurodegeneration, Parkinson’s disease, Alzheimer disease

## Abstract

Neuronal homeostasis depends on the proper functioning of different quality control systems. All intracellular components are subjected to continuous turnover through the coordinated synthesis, degradation and recycling of their constituent elements. Autophagy is the catabolic mechanism by which intracellular cytosolic components, including proteins, organelles, aggregates and any other intracellular materials, are delivered to lysosomes for degradation. Among the different types of selective autophagy described to date, the process of mitophagy involves the selective autophagic degradation of mitochondria. In this way, mitophagy is responsible for basal mitochondrial turnover, but can also be induced under certain physiological or pathogenic conditions to eliminate unwanted or damaged mitochondria. Dysfunctional cellular proteolytic systems have been linked extensively to neurodegenerative diseases (ND) like Alzheimer’s disease (AD), Parkinson’s disease (PD), or Huntington’s disease (HD), with autophagic failure being one of the main factors contributing to neuronal cell death in these diseases. Neurons are particularly vulnerable to autophagic impairment as well as to mitochondrial dysfunction, due mostly to their particular high energy dependence and to their post-mitotic nature. The accurate and proper degradation of dysfunctional mitochondria by mitophagy is essential for maintaining control over mitochondrial quality and quantity in neurons. In this report, I will review the role of mitophagy in neuronal homeostasis and the consequences of its dysfunction in ND.

## Mitochondria and Protein Quality Control in Neurons

Although the origin, progression and heterogeneity of neurodegenerative diseases (ND) differs, these disorders are characterized by common features at the molecular level. These include: (i) accumulation of aggregated misfolded proteins; (ii) impairment of degradative processes including autophagy and the ubiquitin proteasome system (UPS); (iii) oxidative stress; (iv) neuroinflammation; and (v) impaired mitochondrial function comprising mitochondrial dynamics, trafficking and turnover. In this review article, we focus attention on the last-mentioned feature, particularly with respect to impaired mitochondrial degradation by autophagy (mitophagy) in ND.

Due to their metabolic features and post-mitotic state, neurons are particularly vulnerable to mitochondrial dysfunction and to deregulation of the proteostasis system. Mitochondria are the main source of cellular energy, but also have other important cellular functions such as control of programmed cell death, regulation of calcium homeostasis and the biosynthesis of protein cofactors such as heme and iron–sulfur clusters (Nagley et al., 2010; Gleichmann and Mattson, 2011; Stehling and Lill, 2013). Mitochondria form a dynamic network controlled by a range of cellular mechanisms to guarantee an appropriate population of healthy mitochondria for each condition. This mitochondrial network is regulated by the balance between different highly regulated processes: mitochondrial dynamics that control the division (fission) and fusion of mitochondria, *de novo* mitochondrial biogenesis, and the elimination of unwanted mitochondria by mitophagy (Ploumi et al., 2017). Maintaining properly this healthy mitochondrial pool is necessary for cellular homeostasis, but is particularly important for neuronal viability. Neurons have a high energy requirement that is dependent on mitochondrial metabolism as the main energy source. As such, the mitochondrial network needs to be in perfect working order. Moreover, the unique morphology of neurons, with their axons and dendrites, means that mitochondria must be recruited to distant parts of the cell via axonal transport to satisfy energy demands at those sites. Consequently, neurons are vulnerable to defects in axonal trafficking. Finally, due to the post-mitotic state of neurons, cellular division cannot act as a diluting factor for components accumulating within the cell: this means that neurons require an adequate “cleaning system” to eliminate any damaged or unwanted mitochondria that could be a source of reactive oxygen species (ROS) or an inducer of programmed cell death. Given their essential role in neuronal viability, any alterations in mitochondrial function could lead to neuronal death, thus explaining why mitochondrial dysfunction has been linked to numerous ND.

**Figure 1:**
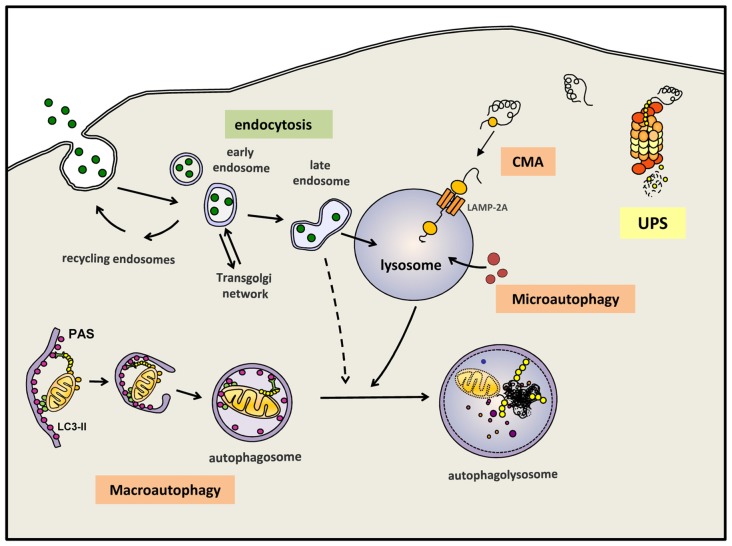
**Autophagy and endocytosis can delivery substrates to lysosome for degradation.** Autophagy is a tightly regulated process by which certain intracellular components are recycled through lysosomal degradation. Various types of autophagy are present in mammalian cells: microautophagy involves the sequestration and degradation of complete regions of the through invaginations of the lysosomal membrane. Macroautophagy (commonly referred as “autophagy”) consists in the engulfment of intracellular components in a double membrane vesicle called autophagosome. This vesicle is then fused with lysosomes to form an autophagoslysosome and the lysosomal hydrolytic enzymes complete the degradation. Under some conditions, macroautophagy can be a selective mechanism where specific substrates are recognized by distinct autophagic receptors and eliminated. Among the different types of selective (macro)autophagy, mitophgay consists in selective degradation of mitochondria. Chaperone-mediated autophagy (CMA) is a proteolytic pathway where specific cytosolic proteins containing a CMA-motif are recognized by chaperones and directly translocated into the lysosome through the CMA receptor formed by LAMP-2A protein. Extracellular components can also be degraded by endocytosis when late endosome fuse with lysosomes or alternatively, first with an autophagosomes and later on with a lysosome. Intracellular proteins can also be degraded by the ubquitin proteasomal system (UPS). All degradative pathways work coordinated and simultaneously to maintain cellular homeostasis.

Autophagy, or more precisely macroautophagy to differentiate it from other forms of autophagy such as chaperone-mediated autophagy (CMA) and microautophagy (Martinez-Vicente, 2015), is the process whereby intracytosolic components, including proteins and/or organelles, are delivered to lysosomes to be degraded (Figure 1). The substrates of this pathway are engulfed inside a double membrane vesicle called autophagosome; once this vesicle is formed and loaded with the substrates to be eliminated, it fuses with a lysosome, a small single-membrane organelle that contains within its matrix many types of degradative hydrolases that can digest the macromolecules into their constitutive components. These components can be reused to build new macromolecules. Autophagy is one of the main mechanism that maintains cellular homeostasis since it is responsible for: (i) the major part of the basal turnover of intracellular material, thus preserving the proper balance between synthesis and degradation; and (ii) a quality control system capable of eliminating defective and/or damaged proteins/organelles generated inside the cell. In addition to autophagy, the cell also contains other intracellular degradative systems as part of its protein quality control system; these alternative mechanisms, working simultaneously and in a coordinated manner with (macro)autophagy, are the UPS and CMA. Whereas the latter systems can only eliminate selected soluble cytosolic proteins, macroautophagy is the only process that can also degrade whole organelles and as well as aggregated proteins. This feature is extremely important, contributing to the essential role of (macro)autophagy in neuron health and survival (Martinez-Vicente, 2015). As mentioned above, neurons are especially vulnerable to any impairment of the degradative systems. To this end, in 2006 the Mizushima and Tanaka groups showed that an active basal autophagy is essential for neuronal viability, as its inactivation leads to the intracytosolic accumulation of protein aggregates and damaged organelles and finally to neuronal cell death (Hara et al., 2006; Komatsu et al., 2006).

In addition to these degradative systems of intracellular components, the endosomal-lysosomal system also contribute to the degradation inside the lysosomes of different types of macromolecules, these substrates provided by endosomes are commonly extracellular components internalized into the cell by endocytosis (Figure 1).

It is possible to differentiate between **basal** autophagy, which is responsible for the continuous turnover of intracellular components, and **induced** autophagy, a stress-response mechanism activated under different conditions to provide essential basic elements like amino acids, to remove damaged proteins or organelles, or to eliminate external pathogens. Thus, induced autophagy can be activated in response to starvation, exposure to oxidative stress, hypoxia, mitochondrial damage, and many other conditions where specific materials must be eliminated from the cell. In fact, autophagy can randomly sequester and eliminate portions of the cytosol with whatever that may contain, in addition to being a selective process to eliminate specific substrates like mitochondria, endoplasmic reticulum (ER), ribosomes, pathogens, lipid droplets, peroxisomes and aggregates (Dunn et al., 2005; Levine, 2005; Bernales et al., 2007; Kim et al., 2007; Kraft et al., 2008; Singh et al., 2009; Yamamoto and Simonsen, 2011) (Figure 1). Although all these types of selective autophagy might be activated by different signals, all such cases follow a similar pattern and require an autophagic receptor, this being a protein that plays a connective role between the substrate and the newly formed or nascent autophagosome.

## Mitophagy

Among the different types of selective autophagy, mitophagy is the field where most research progress has been made in recent times. Over the last decade, knowledge of the molecular mechanisms underlying this process, as well as the proteins and molecules involved, and their role under normal and pathologic conditions has increased greatly.

Mitophagy is the only known pathway via which whole mitochondria can be selectively eliminated. This mechanism was first characterized in yeast in 2004 (Kissová et al., 2004). Later, in 2006, the term “mitophagy” was used by Lemaster’s group in relation to mammalian cells; they showed that depolarized mitochondria localize inside light chain 3 (LC3)-GFP-labeled autophagosomes before being completely degraded (Rodriguez-Enriquez et al., 2006).

Mitophagy is responsible for the **basal** mitochondrial turnover that eliminates old mitochondria once they are no longer needed; however, mitophagy can also be induced under certain **physiological** conditions, examples of which are the maturation of erythrocytes, where mitochondria and other organelles must be removed from the cell, and the development of fertilized oocytes, when paternal mitochondria must be removed (Sandoval et al., 2008; Sato and Sato, 2011). In addition to these physiological roles, mitophagy can be induced as a stress-response mechanism to eliminate selectively damaged mitochondria after depolarization of the mitochondrial membrane or in response to hypoxia. As such, several types of mitophagy have been described, corresponding to the different ways that mitochondria are engulfed within an autophagosome prior to being delivered to a lysosome for completion of the degradation process.

All **types of mitophagy** follow a general pattern involving a receptor-mediated mechanism whereby the receptors physically connect the mitochondria to be eliminated with LC3-II, the main component of the autophagosomal membrane. This connection is established via the LC3-interacting region (LIR) present in all receptors (Wild et al., 2014). The nature and origin of mitophagy receptors can vary depending on the type of mitophagy; some receptors are proteins or lipids localized in the mitochondrial membrane, while others are non-mitochondrial proteins that recognize and simultaneously bind ubiquitinated chains on the mitochondrial surface and LC3-II on the nascent autophagosome structure. These receptors contain both the LIR motif to bind LC3, and the ubiquitin binding domain (UBD) to bind ubiquitin (UB) chains on the targeted mitochondria (Wild et al., 2014).

### Mitophagy Mediated by Mitochondrial Membrane Receptors

**Figure 2:**
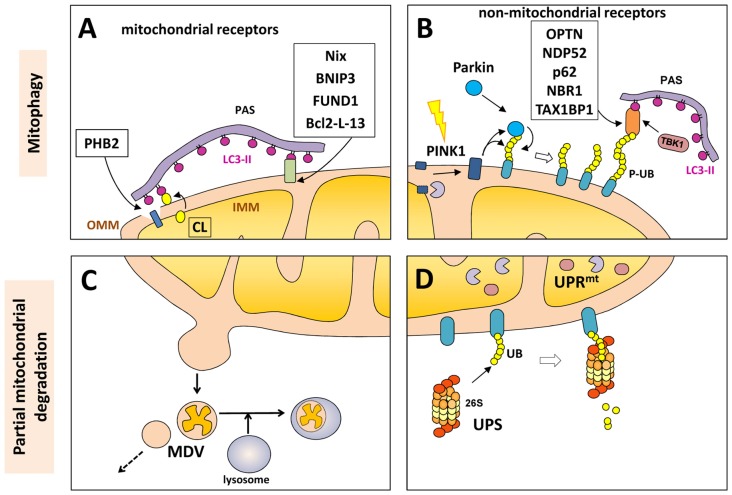
**Mitochondrial degradation.** Whole mitochondria can be degraded by mitophagy (upper panels) under different physiological and stress-induced situations. Different mitophagy receptors link the mitochondria with the pre-autophagosome structure (PAS) through the direct intercation with light chain 3 (LC3)-II (in purple). These receptor can be mitochondrial components **(A)** o non-mitochondrial proteins **(B)** that bind both the phosphorylated ubiquitin (P-UB) chains on the mitochondria surface and LC3-II. Mitochondria can also be partially degraded (lower panel) through the formation of mitochondrial-derived vesicles (MDVs) that can directly be fused with lysosomes **(C)**. Some outer mitochondrial membrane (OMM) proteins can be degraded by the UPS **(D)** after mitochondrial depolarization with the recruitment of the 26S proteasome on the mitochondrial surface. Internally, the mitochondrial unfolded protein response (UPR^mt^) is a quality control system formed by molecular chaperones and proteases **(D)**.

Proteins in the outer mitochondrial membrane (OMM), such as B-cell lymphoma 2 nineteen kilodalton interacting protein 3 (BNIP3), Nix, Bcl-2-like protein 13 (Bcl2-L-13) and FUND1, can act as mitophagy receptors under specific conditions and in certain cell types. All of these proteins contain the LIR motif to facilitate direct interaction of the mitochondria with LC3 or other LC3/GABARAP family members in order to recruit the autophagosomal machinery (Figure 2).

**BNIP3** and its analog **Nix** (also called BNIP3L) belong to the Bcl-2 family and are BH3-only pro-apoptotic proteins. They were initially described as proteins involved in programmed cell death, but later were also shown to act as mitophagy receptors (Zhang and Ney, 2009). Nix was first described as a mitophagy receptor during reticulocyte maturation when mitochondria are eliminated from the erythrocyte (Schweers et al., 2007; Sandoval et al., 2008). BNIP3 and NIX can be regulated by hypoxia, with hypoxia-inducible factor-1 (HIF-1) being a transcriptional factor that activates BNIP3 and NIX expression in response to low oxygen levels. More recently, however, it was shown that these receptors might also be regulated post-translationally by phosphorylation via an as-yet unknown mechanism (Zhu et al., 2013). Another BCL-2 family member, called **Bcl2-L-13**, was recently described, this protein is the mammalian homolog of Atg32, the first mitophagy receptor described in yeast in 2009, this protein can mediate both mitochondrial clearance by mitophagy and also mitochondrial fragmentation (Murakawa et al., 2015).

**FUND1** is another OMM protein that is also activated in response to hypoxia; its activity is regulated by several reversible phosphorylations mediated by different kinases and phosphatases, including ULK1 (Liu L. et al., 2012; Wu et al., 2016).

**AMBRA1** is a multifunctional protein that can act as a sensor to coordinate the cell response to different stressors; its involvement has been described in development, cell proliferation, cell death and autophagy. AMBRA1 can operate as a scaffold protein that regulates the stability of some autophagy-related complexes such as mTOR complex 1 and the ULK1 complex, and can also be partially localized to the mitochondria where it can act as a mitophagy receptor by binding directly to LC3 via its LIR motif (Cianfanelli et al., 2015; Strappazzon et al., 2015).

In 2017, unexpectedly an inner membrane mitochondrial protein has been described as mitophagy receptor. Prohibitin 2 (PHB2) is an inner mitochondrial membrane (IMM) protein that can bind LC3-II through its LIR motif upon mitochondrial depolarization and during the clearance of paternal mitochondria after embryonic fertilization. In both cases, rupture or partial degradation of the OMM mediated by the proteasome is required to expose the inner protein PHB2 to LC3 and the preautophagosome structure (Wei et al., 2017) (Figure 2).

### Cardiolipin-Mediated Mitophagy

Cardiolipin (CL) is a mitochondrial phospholipid present mostly in the IMM. As a component of the mitochondrial membrane, this compound regulates the stability of many mitochondrial membrane protein complexes, like the respiratory chain or the import machineries. It can also modulate other important functions, like mitochondrial dynamics and the induction of apoptosis by regulating the localization of cytochrome c at the IMM (Maguire et al., 2017). Despite being a phospholipid, it also contains an LC3-binding motif and can directly bind the N-terminal domain of LC3-II (Chu et al., 2013). When CL is externalized from the inner to the OMM, it can function as a mitophagy receptor to recruit LC3 and the autophagosome machinery; in this case no protein is involved, the interaction is done directly between CL and LC3-II. The regulation of the externalization of CL is mediated by several proteins (Maguire et al., 2017) and is activated in response to depolarization.

### Non-Mitochondrial Receptors (p62, NDP52, OPTN, NBR1, TAX1BP1) and PINK1/Parkin-Mediated Mitophagy

Mitophagy receptors can also be cytosolic proteins that recognize both the target mitochondria and the autophagosome. Mitochondria are recognized by two domains present in these receptors: the UBD that binds to polyubiquitinated OMM proteins on the mitochondrial surface, and the LIR motif that binds LC3-II present on the autophagosome structure (Wild et al., 2014).

The most appealing and most studied mechanism of this pathway involves how mitochondria are selectively tagged by post-translational modification (phosphorylated-ubiquitin, P-UB chains) for subsequent elimination. In this mechanism, two main proteins, the PTEN-induced putative kinase 1 (PINK1) and Parkin, are involved. This type of mitophagy is known as **PINK1/Parkin-mediated mitophagy** and several groups have contributed to the elucidation of the complex molecular mechanisms by which these two proteins mediate mitophagy in mammalian cells in response to mitochondrial depolarization.

PINK1 has a role as mitochondrial stress sensor; the protein is imported from the cytosol to mitochondria under basal conditions, following which translocase complexes TOM and TIM drive the transport of PINK1 from the OMM to the IMM in a mitochondrial transmembrane potential-dependent manner. Once PINK1 reaches the IMM, it is cleaved by mitochondrial matrix proteases such as mitochondrial processing peptidase (MPP) and presenilin-associated rhomboid-like protease (PARL), with the resulting cleaved protein externalized to the cytosol where it is degraded by the UPS (Jin et al., 2010; Deas et al., 2011; Greene et al., 2012; Yamano and Youle, 2013; Meissner et al., 2015). When damaged mitochondria lose their membrane potential, PINK1 processing is not complete and so full-length PINK1 accumulates in the OMM in association with the TOM complex. This is the first step to trigger the mitophagy of dysfunctional mitochondria (Figure 2).

Once full-length PINK1 is present in the OMM, it can phosphorylates UB at its serine65 tagged to OMM proteins. The presence of P-UB in the OMM stimulates the recruitment and activation of Parkin to damaged mitochondria given Parkin’s high affinity for P-UB. Once at the mitochondrial surface, Parkin is activated by PINK1 kinase activity through the phosphorylation of its serine65. This active Parkin, as an E3 ubiquitin ligase, is then able to promote the ubiquitination of several proteins on the mitochondrial surface that will be subsequently phosphorylated by PINK1, generating a positive feedback loop mediated by PINK1 and parkin. The net result is that damaged mitochondria ends coated by P-UB chains that act as an “eat-me” signal for the mitophagy receptors.

In this model, PINK1 is essential for the phosphorylation of UB and parkin (both at ser 65), while Parkin has an amplifier role, enhancing the presence of the molecular signaling for mitophagy (P-UB) and contributing to the establishment of a more abundant and faster P-UB chain and consequently a stronger mitophagic process.

Recent work by Szargel et al. (2016) showed that, in the absence of Parkin, other E3 UB ligases such as SIAH-1 could catalyze the polyubiquitination of OMM proteins. PINK1 is still necessary for the phosphorylation of UB, however, suggesting that although the P-UB chains on the mitochondrial surface are the signal for mitophagy receptors, parkin is not essential for this process (Szargel et al., 2016).

Parkin can form different types of polyubiquitin chains on different types of OMM substrates. The K48-linked UB chains are the principal signal for UPS degradation, as will be mentioned below, but Parkin can also polyubiquitinate OMM substrates with K63-linked chains; this label is predominantly a tag for autophagy and recognized by different autophagy receptors by means of the UBD domain (Akutsu et al., 2016; Yamano et al., 2016). To date, five different autophagy receptors (p62, NBR1, Optineurin (OPTN), NDP52 and TAX1BP1) are able to recognize polyubiquitinated signals in PINK1/parkin-mediated mitophagy. One of them, p62, has long been linked to selective autophagy (not only mitophagy), being one of the main markers of autophagy substrates and widely used as a marker of autophagic flux together with LC3-II (Klionsky et al., 2016). However, in the recent study by Lazarou et al. (2015) they used different combined knock-outs (KOs) of all five receptors, it was shown that OPTN and NPD52 are indeed the primary receptors for PINK1/parkin-mediated mitophagy. These receptors are recruited to mitochondria by the P-UB signal mediated by PINK1 (which is essential), and enhanced by Parkin (which is important to amplify the signal, but not essential; Lazarou et al., 2015). Accordingly, and as previously proposed, p62 and NBR1 can participate in mitophagy, but are not essential (Narendra et al., 2010). The fact that different receptors can have redundant roles in mitophagy probably suggests the presence of different mitophagy machineries that are called into action depending on the availability of mitophagy receptors for each physiological condition and cell type.

Once the P-UB chains are present and the receptors recruited, an additional step can improve the overall efficiency of the process. TANK binding kinase 1 (TBK1) is a kinase activated by mitochondrial depolarization; it is recruited by the receptors to the mitochondria where it is able to phosphorylate both OPTN and NDP52, thereby enhancing the binding of receptors to UB conjugates (Heo et al., 2015; Richter et al., 2016). The receptors, via their LIR motif, recruit LC3 together with other autophagy machinery members (like ULK1 or Atg9) and the isolated membrane envelops the mitochondria to form an autophagosome.

At this point, there is an additional modulating step, the P-UB chains can be removed by deubiquitinating enzymes (DUBs) that catalyze the removal of ubiquitin chains from substrates. Different DUBs like UPS30, UPS35, UPS8 and UPS15 are able to eliminate the polyubiquitin chains on mitochondria and modulate mitophagy (Bingol et al., 2014; Cornelissen et al., 2014; Durcan and Fon, 2015; Wang et al., 2015). Overexpression of these DUBs can inhibit mitophagy, while siRNA knockdown can activate mitophagy, opening a new therapeutic opportunity to pharmacologicaly induce mitophagy with selective inhibitors of these DUBs.

With the information available at present, we can postulate that PINK1/Parkin-mediated mitophagy uses P-UB conjugates as a molecular signal to trigger selective autophagy. PINK1 is the major player, essential for the phosphorylation of UB, while Parkin plays an important role by amplifying this signal. Once the receptors are recruited, TBK1 can also enhance the interaction between components to achieve a robust mitophagy.

It should be noted that some studies have revealed a role of the **PINK1/parkin machinery in an alternative, mitophagy-independent mechanism** involved in mitochondrial quality control. Several evidences indicate that PINK1 has mitophagy-independent alternative functions, for example, in regulating complex I activity and maintaining neuronal viability in response to stress (Voigt et al., 2016). Parkin activity as an E3 Ub ligase, and its ability to catalyze the polyubiquitination of several mitochondrial proteins is also involved in different mitophagy-independent mechanisms (Sarraf et al., 2013). In this context, numerous reports have linked the role of PINK1 and parkin in **mitochondrial dynamics** because MFN1 and MFN2, two proteins regulating mitochondrial fusion, are polyubiquitinated in a PINK1/parkin-dependent manner upon mitochondrial depolarization. This P-UB serves as a signal for UPS degradation, lower levels of MFN reduce fusion (thus avoiding the fusion of damaged mitochondria with healthy ones) and favores mitochondrial fission (Gegg et al., 2010; Poole et al., 2010; Tanaka et al., 2010; Ziviani et al., 2010; Yoshii et al., 2011). Additionally, other groups described that PINK1/Parkin can also modulate the activity of Drp1, the main regulator of mitochondrial fission (Lutz et al., 2009; Pryde et al., 2016) altogether it seems that the promotion of mitochondrial fragmentation might facilitate its elimination of damaged mitochondria.

PINK1/Parkin may also affect **axonal transport** by phosphorylating and polyubiquitinating Miro, a protein involved in the mitochondrial transport machinery. This labeling of Miro promotes its degradation by the UPS and consequently impairs mitochondrial motility. This arrest/immobilization of damaged mitochondria could prevent their fusion with healthy mitochondria and facilitate its elimination by mitophagy (Wang et al., 2011; Liu S. et al., 2012).

Taken together, PINK1/parkin act in unison to sense mitochondrial depolarization and to label damaged mitochondria, thereby modulating simultaneously different aspects of the mitochondrial quality control system, such as mitochondrial fragmentation, the arrest of mitochondrial motility, and the degradation of damaged mitochondria by mitophagy.

As a last model of selective degradation of mitochondria very recently a new Parkin-dependent pathway for the degradation of whole mitochondria has been proposed by Gustafsson’s lab. In this new form of mitophagy, the damaged mitochondria is recognized in a Parkin-mediated step by the ESCRT machinery of the Rab5-positive early endosome. The ESCRT machinery induces invagination of the endosome membrane, leading to internalization of the mitochondria in the lumen of the endosome. Following maturation, these endosomes deliver mitochondria to lysosomes for degradation (Hammerling et al., 2017). This pathways is similar to the endosomal microautophagy pathway previously described for the elimination of cytosolic proteins inside the endosomes and/or multivesicular bodies (MVB) followed by the delivery to lysosomes for degradation (Sahu et al., 2011) and future work is required to understand the role of this endosomal-mediated mitophagy and the possible redundancy or crosstalk with the autophagsosome-mediated mitophagy.

## Other Forms of (Partial) Mitochondrial Degradation: UPS, UPR^mt^ and MDVs

Although mitophagy is the only cellular process that eliminates whole mitochondria, other mechanisms exist to partially eliminate portions or components of mitochondria. As mentioned above, some OMM proteins can be degraded by the **UPS** after mitochondrial depolarization. This process can be mediated by parkin-dependent polyubiquitination (mostly by K48-linked chains) and recruitment of the 26S proteasome on the mitochondrial surface. This UPS-dependent degradation of OMM proteins (including TOM subunits, PINK1, MFN1, MFN2, VDAC, MIRO and FIS1) precedes mitophagy and is required for proper completion of the mitophagy process (Chan et al., 2011) (Figure 2).

Internally, the mitochondria also have the mitochondrial unfolded protein response (**UPR^mt^**), a quality control system formed by molecular chaperones and proteases that promote proper protein folding, complex assembly, or elimination within the mitochondrial matrix. This stress-response is an early process that activates the transcription of nuclear-encoded mitochondrial genes related to mitochondrial proteostasis as an internal quality control system (Haynes and Ron, 2010).

More recently, a new mechanism involving the partial elimination of portions of mitochondria was described. Mitochondrial-derived vesicles (MDVs) can be formed by budding of the mitochondrial membrane; these vesicles are formed independently of the mitochondrial fission machinery and are targeted to lysosomes for degradation in an autophagosome-independent manner (Soubannier et al., 2012; Roberts et al., 2016) and has some similarities with the mecanim previously described in yeast for the partial degradation of mitochondria (Abeliovich et al., 2013). The MDVs can be formed under basal conditions, but their production is induced in response to oxidative stress. Once again, as part of the global mitochondrial quality control system, PINK1 and Parkin seem to play a regulatory role in the formation of the MDV after mitochondrial depolarization. Some authors proposed that strong depolarization of the mitochondrial membrane will activate the PINK1/Parkin-mediated elimination of mitochondria by mitophagy, while mild mitochondrial damage may promote the PINK1/parkin-mediated removal of a specific portion of the mitochondria through MDVs (Shlevkov and Schwarz, 2014). Although the fate of these vesicles is normally degradation within lysosomes, some MDVs can also be secreted as exosomes or participate in the autoimmune system given the presence of mitochondrial antigens that are presented on major histocompatibility complex (MHC) class I molecules (Matheoud et al., 2016; Figure 2).

## Neuronal Mitophagy and Physiological Vs. Induced Mitophagy

We have thus far reviewed different types of induced mitophagy that occur in response to mitochondrial depolarization, hypoxia or under particular physiological scenarios such as the maturation of reticulocytes or in fertilized oocytes. However, what mechanism is involved in basal mitochondrial turnover in neurons, or in response to mitochondrial dysfunction in neurons? How do neurons, in their condition as post-mitotic cells exhibiting a heightened susceptibility to mitochondrial impairment, manage to selectively eliminate these organelles?

Almost all studies on PINK1/Parkin-dependent mitophagy have been performed on non-neuronal cells, with overexpressed levels of Parkin, and where mitophagy has been induced with the uncoupler CCCP, which induces a rapid and massive loss of the mitochondrial membrane potential in the entire mitochondrial pool. Such conditions (non-neuronal cells, overexpressed Parkin and prolonged CCCP treatment) are not physiological and consequently might not reflect the type of mitophagy occurring in neurons under basal conditions (Grenier et al., 2013).

To this end, different authors have pointed out that neuronal mitophagy might follow a PINK1/parkin-alternative mechanism, principally because the translocation of endogenous Parkin to mitochondria upon depolarization is controversial. Using the methodology typically employed with non-neuronal cells, most groups cannot detect the recruitment of endogenous parkin to mitochondria in neuronal cell lines (Sterky et al., 2011; Van Laar et al., 2011; Cai et al., 2012; Rakovic et al., 2013). Only after the artificially overexpression of parkin, the protein is translocated to mitochondria, activated and mitophagy takes place, however, even under such conditions, the mitophagy process is much slower compared to that seen in non-neuronal cell lines (Cai et al., 2012; Rakovic et al., 2013). Other groups also noted that neurons have a different bioenergetic metabolism and a heavy dependence on oxidative phosphorylation for the production of ATP (compared to the more glycolytic metabolism seen in non-neuronal cells), which might be responsible for the different response of neurons to CCCP and their inability to recruit parkin (Van Laar et al., 2011).

It is known that that mitochondrial turnover is active in neurons (and essential for their survival), meaning that they must somehow manage to eliminate old or damaged mitochondria. In fact, neuronal mitophagy can be measured by quantifying the colocalization of mitochondrial markers with autophagosomes, or lysosomal markers by microscopy analysis (Chu et al., 2013; Klionsky et al., 2016). Neuronal mitophagy can also be quantified by measuring mitochondrial mass in the presence of lysosomal inhibitors; when lysosomal degradation is blocked, an increase of the mitochondrial signal can be detected under basal or induced conditions, corresponding to altered mitochondrial turnover (Mauro-Lizcano et al., 2015).

According to the latest model of Parkin/PINK1-dependent mitophagy, although Parkin might play a role in this pathway, it is not essential. In the presence of low levels of Parkin in neurons, induced mitophagy upon strong depolarization can take place in a PINK1-dependent manner, but the rate of mitochondrial clearance is slow due to the lack of parkin’s amplification. In this context, some authors distinguish between mitochondrial degradation under basal conditions and that in the presence of mild or severe mitochondrial depolarization. Some parkinsonian toxins like rotenone or 6-OHDA can induce mild depolarization in neurons (compared to the strong depolarization with CCCP); such small decreases in mitochondrial membrane potential are not sufficient to activate PINK1/parkin-dependent mitophagy in neurons, and no retention of full length PINK1 or recruitment of parkin can be detected in the mitochondria. However, it has been observed that other alternative pathways, such as that of CL-dependent mitophagy (Chu et al., 2013) can handle the elimination of moderately dysfunctional mitochondria. Alternatively, mild dysfunctional mitochondria could also be eliminated partially by MDV as mentioned above. In summary, neurons can eliminate dysfunctional mitochondria, which is essential for their survival, but different mechanisms might be activated in line with the severity of mitochondrial damage and the cell type involved. In this way, the most well-characterized PINK1/parkin-dependent pathway might be activated in response to severe mitochondrial damage, but this would probably only occur sparingly given the low levels of endogenous parkin present in neurons.

## Mitophagy in Neurodegenerative Diseases

### Parkinson’s Disease

Parkinson’s disease (PD) is the second most common ND after Alzheimer’s disease (AD) and the most common movement disorder. The main risk factor for developing PD is age, affecting about 2% of the population over 60 years. Clinically, PD is characterized by bradykinesia, resting tremor, rigidity and postural instability. At the molecular level, these symptoms are caused by a loss of dopaminergic neurons in the substantia nigra pars compacta (SNpc) and a resulting decrease of dopamine (DA) levels in the striatum. In addition, the disease is characterized by the presence within affected neurons of cytoplasmic protein inclusions called Lewy bodies (LBs), which have alpha-synuclein protein as their main component (Dauer and Przedborski, 2003). Today, despite great advances in biomedical research, only symptomatic treatments are available that relieve many of the motor symptoms of the disease, without stopping or slowing down the progressive death of neurons.

Although most PD patients are diagnosed as sporadic patients with idiopathic PD, other less common forms of familial PD have facilitated the identification of different autosomal recessive and autosomal dominant genes linked to PD, as well as different gene variants identified as PD risk factors (Klein and Westenberger, 2015). Among the monogenic forms of PD, two genes causing autosomal recessive forms of the disease are PARK2 and PARK6, which encode Parkin and PINK1, respectively, and as we have seen above are the main players in PINK1/parkin-mediated mitophagy. The fact that mutations in these two genes are linked to autosomal recessive forms of PD led to greater attention being focused on mitophagy, and the possibility that impaired mitochondrial turnover might be one of the main contributors to PD pathogenesis.

The clinical phenotypes of Parkin- and PINK1-linked PD are indistinguishable from sporadic PD, although the former show an earlier disease onset, particularly in the case of mutated Parkin, which is the most frequent cause of juvenile PD (Matsumine et al., 1997). At the neuropathological level, no apparent differences exist between Parkin- and PINK1-linked PD with sporadic PD in terms of dopaminergic neuronal loss, gliosis and LB load.

Several PINK1-KO and parkin-KO mouse models have been developed in recent years by different groups; however most of them do not recapitulate the PD phenotype. Most of these animal models do not show neuronal loss, altered DA metabolism, presence of LBs, or abnormalities in motor behavior (Blesa and Przedborski, 2014). These PINK1- and parkin-KO mice, however, exhibit a clear mitochondrial dysfunction, but probably not enough to trigger neuronal death. Only some particular mouse models developed by some groups exhibit a slight impairment of DA release (Itier et al., 2003; Kitada et al., 2009) and some knock-in mutant Parkin mice present some minor motor deficits and neuronal loss (Sriram et al., 2005; Van Rompuy et al., 2014). In contrast, parkin- and PINK1-mutant fly models have shown a more clear-cut PD phenotype, including mitochondrial dysfunction as well as dopaminergic neuronal loss, significant motor disabilities and reduced lifespan (Greene et al., 2003; Pesah et al., 2004; Clark et al., 2006; Yang et al., 2006; Burman et al., 2012).

These results again suggest that PINK1 and parkin play important roles in the mitochondrial quality control system of neurons, including but not limited to mitophagy. Due to the particular vulnerability of neurons to mitochondrial impairment, any alteration of the mitochondrial homeostasis might contribute the pathogenesis of PD. In the mouse models, damage to the mitochondria might not be sufficient to affect neuronal viability, or other processes might compensate for the PINK1/parkin loss. In humans, however, the occurrence of mutations in these genes might be enough to trigger PD.

### Alzheimer’s Disease

AD is the most common neurodegenerative disorder, resulting in severe memory loss and cognitive dysfunction. The principal pathological hallmarks of AD are intracellular neurofibrillary tangles (NFTs), mostly constituted by hyperphosphorylated Tau protein, and extracellular senile plaques containing amyloid-β peptide (Aβ), derived from proteolytic processing of amyloid precursor protein (APP; Ittner and Götz, 2011).

Increasing evidence shows that, in the etiology of AD, different processes contribute to neurodegeneration, including mitochondrial dysfunction, oxidative stress and impairment of the autophagic/lysosomal/endosomal system. Numerous studies have shown that alterations in mitochondrial dynamics, morphology, motility and activity are linked to AD, and together with oxidative stress seem to be important early events in this disease (Nunomura et al., 2001; Moreira et al., 2010; Cai and Tammineni, 2016).

On the other hand, work done by Nixon’s group and others have shown that the lysosomal network, including the two pathways that deliver material to the lysosome (autophagy and the endosomal system), are affected in AD. Ultrastructural analyses of AD brains have revealed that affected neurons exhibit abnormally enlarged endosomes, depleted lysosomes, and accumulation of autophagosomes loaded with undigested material that cannot be cleared by lysosomes (Nixon et al., 2000, 2005; Nixon and Yang, 2011; Ihara et al., 2012).

The formation of extracellular senile plaques containing Aβ starts with the processing of APP by β- and γ-secretases; some of the resulting Aβ peptides can spontaneously aggregate to form oligomers and subsequently insoluble fibrils in a process called amyloidogenesis (LaFerla et al., 2007). These oligomeric Aβ are the precursors of the insoluble amyloid fibrils and are considered responsible for the neurotoxicity present in AD. This amyloidogenic processing can occur both externally at the plasma membrane, where APP localizes, as well as inside the cell, in the membranes of different cellular compartments where the APP along with the β- and γ-secretases are also present. These intracellular compartments include endosomes, the trans-Golgi network (TGN), the ER, lysosomes, autophagosomes, MVB and mitochondria (Walsh et al., 2002). Thus, Aβ can be found both extracellularly and intracellularly where it can interfere with different cellular pathways. The endosomal system participates at different levels in the production of Aβ. These include the processing of APP to Aβ at the endosomal membrane, the internalization of external Aβ by endocytosis, and in the intracellular trafficking and secretion of Aβ to the outside of the cell. Changes in the endosomal system have been observed in AD, and several proteins regulating this pathway shown to be altered and thereby affecting the regulation, sorting, trafficking and secretion of endosomal vesicles. As a result, levels of intracellular Aβ increase and promote further endosomal dysfunction as a consequence (Peric and Annaert, 2015).

The acidification of lysosomes can be compromised when PS-1 (which causes early-onset familial AD) is mutated or absent, because the processing of the subunits of the lysosomal pump is affected. As a result, lysosomal proteolysis is impaired and lysosomes cannot degrade the material loaded by endosomes or autophagosomes (Lee et al., 2010). Lysosome stability can also be affected by the presence of the lipoprotein ApoE4 (whose gene mutation is the main genetic risk factor for AD) and Aβ, both of which can bind to and destabilize the lysosomal membrane and lead to lysosomal membrane permeabilization (Ji et al., 2006). As a compensatory response, several genes promoting autophagy are up-regulated at the transcriptional level (Lipinski et al., 2010); however, ultimately, there is an accumulation of abnormal endosomes and overloaded autophagosomes.

In summary, AD-associated dysfunction is linked to the endosomal pathway, lysosomal integrity and the autophagic pathway, all of which are inter-related and -dependent. However, although selective autophagy (and intrinsically, mitophagy) might be affected in AD, very little has been studied on this topic, and only a few reports have focused on the role of mitophagy in AD (Khandelwal et al., 2011; Wang et al., 2016). Since mitochondrial dysfunction is a hallmark of AD, and neuronal autophagy is also dysfunctional in affected neurons, we suggest that mitophagy might also be impaired. It remains to be determined if the presence of dysfunctional mitochondria is a consequence of inefficient mitophagy, or whether mitochondrial dysfunction is an early event preceding impairment of the autophagy/mitophagy systems.

### Amyotrophic Lateral Sclerosis

Amyotrophic lateral sclerosis (ALS) is a fatal disease characterized by the selective degeneration of motor neurons. A hallmark of the disease, as in other ND, is the accumulation of aggregated proteins within affected neurons. Most ALS cases are sporadic (SALS), with only about 10% classified as familial forms of ALS (FALS). Genetic studies of these familial cases has permitted the identification of several genes linked to ALS (Cirulli et al., 2015). Among these are several genes related to cellular quality control pathways, and more specifically to selective autophagy and mitophagy. These include genes encoding the mitophagy receptors OPTN and p62 (or SQSTM1), as well as TBK1, which activates mitophagy receptors via phosphorylation. *In vitro*, different ALS-linked mutations in genes encoding these proteins can affect selective autophagy. In this way, mutant OPTN and TBK1 can interfere with the process of mitophagy, while mutant p62, which shows a lower affinity for LC3-II, reduces the efficiency of selective autophagy (Maruyama et al., 2010; Wong and Holzbaur, 2014; Majcher et al., 2015; Moore and Holzbaur, 2016). These data suggest that the inefficient turnover of damaged mitochondria and also aggregates, may contribute to neurodegeneration in ALS.

Frontotemporal dementia (FTD) is an ND affecting cortical neurons and the basal ganglia, resulting in cognitive impairment, language deficiency and changes in social behavior and conduct. Despite the differences in the types of neurons affected, and consequently in the clinical symptoms manifested in ALS and FTD, both diseases show significant clinical and genetic overlap (Weishaupt et al., 2016), with up to 50% of patients with ALS developing FTD symptoms and approximately 15% of FTD patients displaying motoneuron dysfunction typical of that seen in ALS (Lomen-Hoerth et al., 2002). Some of the genes linked to ALS were also found to be related to FTD, among which are those related to mitophagy/selective autophagy: OPTN, TBK1 and p62. The molecular mechanisms underlying which neurodegenerative disorder (ALS, FTD or ALS/FTD) is manifested in the presence of mutations in these genes remain unknown.

### Huntington’s Disease

Huntington’s disease (HD) is an autosomal-dominant neurodegenerative disorder caused by an expansion of the cytosine-adenine-guanine (CAG) trinucleotide repeat encoding a polyglutamine (polyQ) tract in the amino-terminal region of Huntingtin (Htt) protein. Clinically, HD is characterized by motor dysfunction, cognitive decline and psychiatric disturbances caused by the preferential atrophy of GABAergic medium spiny neurons in the striatum as well as in other regions such as the cerebral cortex (Ross and Tabrizi, 2011).

The expansion of more than 37 CAG repeats within the huntingtin gene generates an aberrant, misfolded protein (mutant Htt or mHtt) that is prone to form aggregates in the affected neuron (Sapp et al., 1997). Htt is a huge protein that can interact with numerous other proteins (Li and Li, 2004; Kaltenbach et al., 2007; Culver et al., 2012), meaning that multiple biological functions can be affected in the presence of mHTT. Several physiological mechanisms have been implicated in the HD pathogenesis, however the complete etiology of this disease remains unclear.

Among many cellular mechanisms, mHTT has been proposed to affect transcriptional regulation, synaptic transmission, axonal trafficking, nuclear export/import, ubiquitin-mediated proteolysis and regulation of the endosomal/autophagic system (Pal et al., 2006; Martinez-Vicente et al., 2010; Caviston et al., 2011; Koga et al., 2011; Costa and Scorrano, 2012; Gouarné et al., 2013; Guedes-Dias et al., 2016). As in other ND, mitochondrial dysfunction and proteostasis alterations, particularly in the autophagic/endosomal system, are linked to the pathogenesis of HD (Ross and Tabrizi, 2011).

The role of mHTT in the pathogenesis of the autophagic/lysosomal/endosomal system has been described at multiple molecular steps; in the initiation of the autophagic process, mHtt can affect the expression at the transcriptional level of different autophagy-related genes (Metzger et al., 2013). At the cargo recognition step in selective autophagy, the Htt protein plays an important role as a scaffolding protein in the stabilization of the different protein complexes involved. The presence of the polyQ tract can affect the efficiency of this step and consequently the clearance of damaged mitochondria by mitophagy (Martinez-Vicente et al., 2010; Ochaba et al., 2014; Rui et al., 2015). Htt protein can also interact with motor proteins like dynein and kinesin, thus playing an important function in the microtubule-dependent transport of endosomes, autophagosomes and lysosomes. The presence of mHtt can affect the efficiency of all the pathways that strongly depend on vesicular trafficking like autophagy and endocytosis to which we should also add the effect of mHtt on different Rab proteins, the regulators of the endocytic trafficking (Caviston and Holzbaur, 2006; Caviston et al., 2007, 2011; Ravikumar et al., 2008).

Beyond HD, there are other polyQ diseases characterized by the pathological expansion of CAG trinucleotide repeat. Despite the fact that in each polyQ disorder a different protein carries the polyQ tract, all these disorders present some common molecular mechanisms related to the pathology like the aggregation of the mutant protein, alterations of the protein quality control including autophagy and mitochondrial dysfunction (Weber et al., 2014).

## Conclusion

Since 2006 it has been well established that a lack of basal autophagy in neurons, including all types of selective macroautophagy such as mitophagy, is enough to produce neuronal death (Hara et al., 2006; Komatsu et al., 2006). The neuronal-specific Atg5-KO and Atg7-KO mouse models demonstrated that if neurons in the central nervous system cannot properly turnover proteins and organelles like mitochondria the consequence is the accumulation of polyubiquitinated substrates and inclusion bodies, resulting in cell death. Thus mitophagy is essential for neurons, under basal conditions and after mitochondrial damage, and neurons seem to be able to eliminate partially or completely mitochondria through the action of different mechanisms. Whether these mechanisms are coordinated or redundant, or whether they are activated in response to different conditions is an issue that stills requires future investigations, but undoubtedly dysfunctional mitophagy in neurons might have important implication in neurodegeneration.

Regarding PINK1/parkin-mediated mitophagy role in neurons, we must consider this pathway as one of the possible mitophagy pathways used by neurons to eliminate mitochondria after a strong depolarization, however, we must be aware that the participation of endogenous Parkin in neuronal mitophagy might not be such essential as the observed in artificial cellular models. However, we should have a boarder view of PINK1 and Parkin as mitochondrial stress sensors and key components of the global mitochondrial quality control system and not only in mitophagy.

## Author Contributions

The author confirms being the sole contributor of this work and approved it for publication.

## Conflict of Interest Statement

The author declares that the research was conducted in the absence of any commercial or financial relationships that could be construed as a potential conflict of interest.
